# Feedback and coaching

**DOI:** 10.1007/s00431-021-04118-8

**Published:** 2021-05-21

**Authors:** Adelle Atkinson, Christopher J. Watling, Paul L. P. Brand

**Affiliations:** 1 Division of Immunology and Allergy, Department of Paediatrics, Hospital for Sick Children, University of Toronto, Toronto, Canada; 2 Centre for Education Research and Innovation, Schulich School of Medicine and Dentistry, Western University, London, Canada; 3 Department of Medical Education and Faculty Development, Isala Hospital, Isala Academy, Zwolle, the Netherlands; 4 Lifelong Learning Education and Assessment Research Network (LEARN), University of Groningen, Groningen, the Netherlands; 5 University Medical Centre, Groningen, the Netherlands

**Keywords:** Feedback, Coaching, Assessment, Medical education

## Abstract

If used thoughtfully and with intent, feedback and coaching will promote learning and growth as well as personal and professional development in our learners. Feedback is an educational tool as well as a social interaction between learner and supervisor, in the context of a respectful and trusting relationship. It challenges the learner’s thinking and supports the learner’s growth. Coaching is an educational philosophy dedicated to supporting learners’ personal and professional development and growth and supporting them to reach their potential. In clinical education, feedback is most effective when it is explicitly distinguished from summative assessment. Importantly, feedback should be about firsthand observed behaviors (which can be direct or indirect) and not about information which comes from a third party. Learners are more receptive to feedback if it comes from a source that they perceive as credible, and with whom they have developed rapport. The coaching relationship between learner and supervisor should also be built on mutual trust and respect. Coaching can be provided in the moment (feedback on everyday clinical activities that leads to performance improvement, even with short interaction with a supervisor) and over time (a longer term relationship with a supervisor in which there is reflection on the learner’s development and co-creation of new learning goals). Feedback and coaching are most valuable when the learner and teacher exhibit a growth mindset. At the organizational level, it is important that both the structures and training are in place to ensure a culture of effective feedback and coaching in the clinical workplace.

*Conclusions*: Having a thoughtful and intentional approach to feedback and coaching with learners, as well as applying evidence-based principles, will not only contribute in a significant way to their developmental progression, but will also provide them with the tools they need to have the best chance of achieving competence throughout their training.

**Table:** 

**What is Known:** *• Feedback and coaching are key to advancing the developmental progression of trainees as they work towards achieving competence.* *• Feedback is not a one-way delivery of specific information from supervisor to trainee, but rather a social interaction between two individuals in which trust and respect play a key role.* *• Provision of effective feedback may be hampered by confusing formative (supporting trainee learning and development) and summative (the judgment that is made about a trainee’s level of competence) purposes.*	
**What is New:** *• Approaches to both the provision of feedback/coaching and the assessment of competence must be developed in parallel to ensure success in clinical training programs.* *• Faculty development is essential to provide clinical teachers with the skills to provide effective feedback and coaching.* *• Coaching’s effectiveness relies on nurturing strong trainee-supervisor relationships, ensuring high-quality feedback, nourishing a growth mindset, and encouraging an institutional culture that embraces feedback and coaching.*

**What is Known:**

*• Feedback and coaching are key to advancing the developmental progression of trainees as they work towards achieving competence.*

*• Feedback is not a one-way delivery of specific information from supervisor to trainee, but rather a social interaction between two individuals in which trust and respect play a key role.*

*• Provision of effective feedback may be hampered by confusing formative (supporting trainee learning and development) and summative (the judgment that is made about a trainee’s level of competence) purposes.*

**What is New:**

*• Approaches to both the provision of feedback/coaching and the assessment of competence must be developed in parallel to ensure success in clinical training programs.*

*• Faculty development is essential to provide clinical teachers with the skills to provide effective feedback and coaching.*

*• Coaching’s effectiveness relies on nurturing strong trainee-supervisor relationships, ensuring high-quality feedback, nourishing a growth mindset, and encouraging an institutional culture that embraces feedback and coaching.*

## Clinical teaching scenario

You are supervising Janice, a 1st year pediatric resident, who has just completed her evaluation of an 11-month old infant referred for concerns about potential gross motor developmental delay. After the encounter, Janice takes a moment to organize herself and then presents the case to you. The first thing you notice is that while her case presentation does focus on gross motor development, she did not ask the parents about milestones in the infant’s other important developmental domains and they were not included as part of her examination. You have no previous experience with Janice. How do you approach the feedback conversation which you are going to have with Janice about her evaluation of this patient?

## Definitions

Used thoughtfully and with intent, feedback and coaching can accelerate clinical learning. For their potential to be realized, we need to be clear about what we are doing and why (Table 1). Traditionally, feedback has been defined as information that allows learners to compare their actual performance with that of a standard to which they aspire, *and* that enables them to take action to remedy the gap between the two [1, 2]. More recent definitions of feedback highlight its fundamentally social nature, framing it as a “dynamic and co-constructive interaction in the context of a safe and mutually respectful relationship for the purpose of challenging a learner’s (and educator’s) ways of thinking, acting or being to support growth” [3]. This more nuanced definition encourages us to think of feedback as a conversation and not a simple transaction.

**Table 1:** Definitions of feedback, coaching, and assessment

Feedback: - *Content*: information allowing learners to compare their actual performance with that of a standard to which they aspire, *and* that enables them to take action to remedy the gap between the two. - *Relationship*: dynamic and co-constructive interaction in the context of a safe and mutually respectful relationship for the purpose of challenging a learner’s (and educator’s) ways of thinking, acting, or being to support growth.	
Coaching: An educational philosophy dedicated to helping learners to realize their potential, emphasizing learning, performance improvement, and personal or professional growth. - Coaching in the moment: daily coaching interactions between learner and supervisor leading to the learner’s performance improvement - Coaching over time: longitudinal relationship between a learner and a mentor, promoting the learner’s reflection on their performance and formulation of new learning objectives	
Assessment: A judgment of the quality of a learner’s performance - Assessment for learning: formative use of assessment to fuel conversations that help learners to fine-tune knowledge and skills - Assessment of learning: summative judgment of a learner’s performance compared against a standard	
Growth mindset: A motivation theory proposing that implicit mindsets are situated along a *continuum* with two extremes. At one end of the continuum are individuals with a fixed mindset who believe intelligence and ability are static, and talent is something you are born with. Individuals with a growth mindset believe these traits are malleable and effort can lead to success. As individuals, our mindset can vary along the continuum depending on a number of factors including age and context [33].	

Coaching and feedback intertwine, so it is useful to consider how they relate to one another. While feedback is an educational tool, coaching is an educational *philosophy* dedicated to helping learners to realize their potential (box 1) [4]. Coaching emphasizes learning, performance improvement, and personal or professional growth [5]. Coaches use feedback as an essential tool of the trade, but their work encompasses a number of other elements, including direct observation, targeted goal-setting, and habits of reflection. In medical education, the relationships between coaches and learners are inherently hierarchical, which is a potential barrier for learners to show doubts or concerns. Reducing this hierarchy is no easy feat. Longitudinal coach-learner relationships built on trust can help to ensure that the vulnerability required for meaningful performance improvements feels safer for learners [4].

## Feedback or judgment?

Assessment has sometimes been positioned as the enemy of coaching [6], but in fact is an essential partner. To assess is to judge the quality of a learner’s performance (Table 1). A sound understanding of a learner’s strengths and weaknesses is a necessary foundation for both constructive feedback and effective coaching. But we must remain clear-eyed about the intent of our assessment when engaging in feedback and coaching. While assessment may be used formatively to fuel conversations that help trainees fine-tune skills (feedback and coaching), assessment may also be used summatively, to make more consequential judgments that compare trainees against a standard [7]. Comparing trainees against the minimally acceptable level for future doctor performance is important because society expects the medical education community to ensure that trainees who fail to attain these minimal standards are not allowed to obtain a license to practice medicine [8, 9]. This formal high-stakes judgment at the end of a learning period (“assessment of learning”) will be addressed in a separate paper in this series. In the present article, we will focus on how clinical supervisors can provide feedback and coaching to help trainees improve their knowledge, skills, and competence as a junior doctor (“assessment for learning”). Although the importance of making this distinction has been recognized for over 25 years [10], mixing up feedback and summative judgments remains common [11]. This is undesirable because it reduces the usefulness and effectiveness of feedback [12, 13].

## Conditions for effective feedback

There is good evidence that feedback can be highly effective [14–16]. Feedback from their supervisors helps learners at all stages of medical education to make the most of the experiential learning opportunities in encounters with patients. Learners are willing and able to change their behavior consistently based on constructive feedback, and this helps them to work towards practicing independently, without supervision [17]. However, feedback can only be expected to be effective if it meets certain conditions (Table 2) [16, 18, 19].

**Table 2:** Conditions for effective feedback

Aimed at improvement and growth, not at judgment or disapproval Based on direct observation of task Task-oriented (not person-oriented) Respectful approach Constructive, offering an action plan for progress (actionable) Credible source Dialogue with input from both learner and supervisor Reasonable amount	

Feedback is the main technical component of coaching, a momentary tool for purposeful reflection on a concrete learning situation. Feedback is most likely to help learners change their behavior if it is delivered in a constructive and actionable fashion, aimed at the task that the learner has performed. Feedback should be about observed behaviors, not about rumors or indirect information [20]. Learners are more receptive to feedback if it comes from a source that they perceive as credible [14]. Presenting feedback in a dialogue between learner and supervisor instead of a one-way transfer of information from supervisor to learner acknowledges the social and emotional intricacies of human relationships, which also supports acceptance of the feedback provided, and acting upon it [21]. This is also supported by limiting the amount of feedback to one to three key points of the observed task.

The key distinction between effective and ineffective feedback lies in its aim. Medical students and residents are less receptive to feedback if it is presented or perceived as a summative judgment of their performance [13]. They just want to pass the test and be reassured that they did a good job, and tend to ignore or discard the feedback associated with these high-stakes assessments [22, 23]. Residents who perceive workplace-based assessments as high-stakes exams tend to “play the game” of seeking only positive feedback (i.e., only ask for feedback on a task or procedure they think they did well) [22, 24]. Residents may employ this and other impression management strategies to portray an image of competence [24, 25]. Conversely, medical students and residents are considerably more receptive to feedback when it is presented as a low-stakes learning opportunity. Particularly, when it is framed as repeated coaching over time aimed at improving clinical skills, constructive feedback from skilled and dedicated supervisors is likely to be accepted by the learners and acted upon, especially against the background of established rapport that evolves from the working relationship [26].

It is like driving lessons and driving tests: people are receptive to feedback during driving lessons (they are in learning mode) but not during the high-stakes driving test when they just want to pass the test and get their driver’s license (exam mode) [13]. If you want your medical students and residents to accept and act upon your feedback, present it as a low-stakes learning opportunity in which you are there to support and help, not judge, the learner. This requires the supervisor to take the time and the effort to agree on the aim of the coaching session, to observe the learner, encourage reflection by the learner, and discuss the feedback. This becomes easier and less time-consuming with an evolving constructive working relationship built on mutual trust and respect.

## Using feedback in workplace-based coaching

The notion of formative feedback and coaching in a low-stakes environment, and the evidence of its effectiveness in learner growth and development [8–10], is core to the concept of competency-based medical education (CBME) [27]. Workplace-based assessment in the context of CBME includes (preferably direct) observations in the authentic clinical environment, coaching and feedback, and documentation of some of the interactions. Providing workplace-based assessments on a daily basis supports the low-stakes approach of each of these feedback conversations and provides repeated opportunities for the learner to incorporate the coaching into their practice.

Data from workplace-based assessments accumulated in a portfolio, along with other information about the learner’s professional performance, will at some timepoint be reviewed, to make a determination about a trainee’s progress in the program and design an appropriate learning plan (see the paper on assessment in this series). Each workplace-based assessment (data point) completed for a learner will contribute to the overall assessment of their development and progression. In CBME, it is essential that the goal of assessing competence and the goal of providing ongoing developmental coaching and feedback to the learner are intentionally developed in parallel [13]. This requires that both learners and supervising faculty have a shared mental model of the overarching goals of the coaching and assessment program.

## Feedback and coaching in the social relationship between trainee and coach

In order for feedback to be effective in coaching, one must consider a few important concepts: the coaching relationship (between trainee and coach); the quality of the coaching interaction; the mindset of the trainee who is receiving the coaching; and the organizational culture around coaching.

### Relationship

The coaching relationship between trainee and coach is important and complex. Trainees and coaches find themselves in a variety of contexts together, each of which must be considered when designing an intentional plan to support the learner’s growth within a positive learning environment. One way to think about these different contexts has been articulated as “coaching in the moment” and “coaching over time” (see Table 1) [28, 29]. Coaching in the moment describes those day-to-day interactions between a trainee and a clinical supervisor that lead to performance improvement. Coaching over time refers to a more longitudinal faculty-trainee relationship outside the clinical environment, in which the educational partnership promotes the trainee’s reflection on his or her performance, the accumulated data, and a co-developed plan for ongoing development. The main goal of coaching over time is to support learners in their progress as medical professionals, and to help them set new learning objectives. A supportive relationship between coach and trainee supports the value of coaching and helps to design the path forward around the acquisition of knowledge, skills, and attitudes [13]. Successful coaching over time may also help learners to develop the attitudes and skills necessary for ongoing, self-critical reflection, and thus for life-long learning and openness to feedback. It is likely that this will have a positive impact on the quality of care, the effectiveness of collaboration, and personal motivation.

### Quality of the coaching interaction

Coaching in the moment may be powerful, even in large departments with fragmented supervision and lack of trainee-supervisor continuity [30]. But effectiveness rests on ensuring that each coaching interaction is meaningful. Coaching is not always intuitive, and faculty development (training supervisors in providing effective feedback and coaching) is needed to ensure clinical faculty understand their roles and are provided with the tools needed to perform this task effectively [31].

The R2C2 mnemonic is a useful model for feedback interactions in coaching (Table 3), and offers a framework for this necessary faculty development. It focuses on four phases that ultimately lead to a high-quality, two-way feedback and coaching conversation, and it attends carefully to the key role of the supervisor-trainee relationship [32]. This model urges the supervisor to build rapport with the trainee and explore his or her reactions to feedback, ensuring a shared (formative) aim of the interaction, before addressing the specific content of the feedback, and coaching for change (performance improvement). This model for coaching interactions can be used both for coaching in the moment and for coaching over time.

**Table 3:** The R2C2 model for effective feedback and coaching [32]

R: build rapport with the trainee R: explore reactions to feedback C: explore content of feedback by discussing what went well and what can be improved C: coach for change: discuss how the learner can grow in skills and competence	

### Mindset of the trainee

The receptiveness of trainees to coaching feedback is key to the success of any coaching interaction. This raises the notion of where a learner’s mindset might sit at any given moment, on the continuum between a fixed and a growth mindset. Individuals with a predominantly fixed mindset believe that intelligence, ability, and talent are fixed, while those with a growth mindset believe these traits can be influenced with effort. What is clear, however, is that there are strategies to move an individual in the direction of a growth mindset which allows coaching feedback to fall on fertile ground [33]. Individuals with a predominantly growth mindset are learning-oriented instead of performance-focused, and value ongoing skill development [17]. They are open to coaching feedback, making an effort to incorporate it into their practice to continue to develop, a skill that is invaluable throughout their careers as a life-long learner. Having a growth mindset also helps trainees to focus on the formative nature of the coaching feedback, rather than the summative nature of assessment data interpretation.

### Organizational culture around coaching

A growth mindset culture can be nurtured with specific strategies targeted at the trainee, the supervisor, and the organization. For example, having an explicit session on growth mindset during resident orientation has been shown to facilitate the coaching feedback process [33]. Supervisors need to provide the psychological safety to ensure that learners can be open and vulnerable about their challenges and weaknesses and seek feedback for growth [34]. Clinical faculty are uniquely poised to role model this behavior by discussing their own weaknesses and uncertainties, and by being overtly receptive to feedback themselves [35]. At an organizational level, the promotion of a safe and just culture that promotes feedback at all levels is essential [33]. In addition, providing the physical space to encourage and support direct observation, and developing a culture that nurtures essential trusting relationships between trainees and supervisors, is paramount to successful coaching.

The goals and structure of the educational program, and the role workplace-based assessments and coaching play in it, should be clear to both faculty and learners. In addition, supervisors should be trained to develop the skills to provide effective feedback in coaching in the moment and coaching over time. A program in which both faculty and trainees express a growth mindset and are open to provide and receive feedback regularly is most likely to support the learners’ development towards the desired level of clinical competence [35].

## Clinical teaching scenario—part 2

As we return to our junior resident Janice in our original scenario, how could we approach this interaction from a coaching and feedback perspective? You start by taking a moment to build rapport to enable the coaching conversation, for example by asking her how she is enjoying her outpatient clinic experience so far. You reassure her that the present coaching conversation is intended to help her grow and develop, and is not a summative assessment of her competence. Inviting her own reflections on the clinical interaction will allow you to assess her content knowledge and her level of insight. You can start this piece by asking what she thinks went well, and then add or confirm the things that you felt went well. Beginning with what went well tends to boost learners’ confidence and makes them more receptive to hear points for improvement. Next, when you ask her to reflect on what can be improved, she may realize herself that she has omitted the other developmental domains, and articulate that she feels disorganized in her approach. This will help you frame the next part of the coaching conversation, in which you can coach her to address the omission around the developmental milestones, either from a knowledge or an organizational perspective, articulating why they are important in this particular clinical scenario. It is important to address not only the issue of what can be improved, but also how to accomplish this. Providing some practical tips on how to organize and frame the questions to the caregiver, and how to perform the physical examination maneuvers on a child of this age, would be helpful for her skill development moving forward. You can then ask her to summarize what she takes away from the coaching conversation, ask her to record this in her portfolio, arrange to observe her in your next clinic together, and commit to continuing the conversation at that time.

## Conclusion

Effective feedback and coaching are essential to learner development, progression, and achievement of competence. With mindful attention to evidence-based principles, the energy put into these activities will yield the positive results needed to support our learners and ensure their success.

## Data Availability

N/A

## Abbreviation

CBME: Competency-based medical education

## Code availability

N/A
